# Safety, toxicity and pharmacokinetic assessment of oral Withaferin-A in mice

**DOI:** 10.1016/j.toxrep.2022.05.012

**Published:** 2022-05-18

**Authors:** Saurabh Kumar Gupta, Shraddha Jadhav, Dievya Gohil, Girish Ch. Panigrahi, Rajiv Kumar Kaushal, Khushboo Gandhi, Anand Patil, Preeti Chavan, Vikram Gota

**Affiliations:** a Department of Clinical Pharmacology, ACTREC, Tata Memorial Centre, Kharghar, Navi Mumbai 410210, Maharashtra, India; b Department of Pathology, Tata Memorial Hospital, Parel, Mumbai 400012, Maharashtra, India; c Composite Laboratory, ACTREC, Tata Memorial Centre, Kharghar, Navi Mumbai 410210, Maharashtra, India; d Homi Bhabha National Institute, BARC Training School Complex, Anushakti Nagar, Mumbai 400094, Maharashtra, India

**Keywords:** Withaferin-A, Acute toxicity, Sub-acute toxicity, Pharmacokinetics, No-Observed Adverse Effect Level (NOAEL), *Withania somnifera*

## Abstract

Withaferin-A (WA) is the principle component of *Withania somnifera* (Ashwagandha). It has several biological activities including anti-cancer, anti-diabetic, neuroprotective, hepatoprotective and immune-modulatory properties. The acute and sub-acute toxicity of oral WA was investigated in mice. In the acute toxicity study, up to 2000 mg/kg of WA was well tolerated without any signs of toxicity or death. In the sub-acute toxicity study, mice were orally administered 10, 70 and 500 mg/kg of WA respectively, daily for 28 days. Upon physiological, serum chemistry, hematology and histopathogical examination, no features suggestive of drug-induced toxicity were observed at any dose levels, thereby confirming the No-Observed Adverse Effect Level (NOAEL) to be at least 500 mg/kg. Furthermore, the oral bioavailability of WA was evaluated using single intravenous and oral doses of 10 mg/kg and 70 mg/kg respectively using sparse sampling strategy. Bioanalysis was carried out using a validated LC-MS/MS method. The AUC of WA was found to be 3996.9 ± 557.6 ng/mL*h and 141.7 ± 16.8 ng/mL*h for the intravenous and oral routes of administration respectively. The oral bioavailability was determined to be 1.8%. To conclude, WA was found to be extremely safe even at high doses, with a low oral bioavailability.

## Highlights

• In acute toxicity study Withaferin-A was found to be safe up to 2000 mg/kg.
• The observed LD_50_ of Withaferin-A was > 2000 mg/kg body weight.
• Based on repeat dose toxicity 500 mg/kg of Withaferin-A was considered as NOAEL dose.
• The oral bioavailability of Withaferin-A was found to be 1.8%.

## Graphical Abstract

**Figure:**
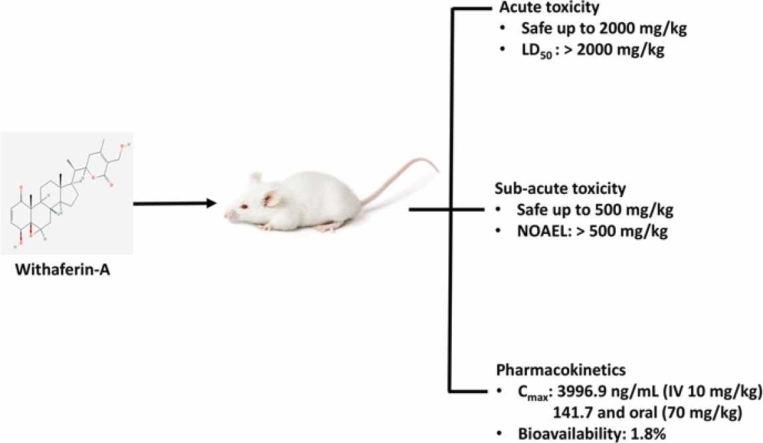

## 1 Introduction

Plants have served as a source of medicine over 4000 years [1]. The biologically active phytochemicals have fashioned a cure for many illnesses and are actively pursued by the pharmaceutical industry [2]. Their therapeutic benefit against communicable (viral & bacterial) and non-communicable (cancer, diabetes, autoimmune disorders etc.) diseases are well documented [3], [4], [5]. Systematic scientific research on plant based medicine started in the 1950s [6]. As per the WHO estimates, approximately 80% of the population of the world seek health benefits from herbal drugs [7]. Among all ancient culture, India represents the richest repository of medicinally valued plants [8]. One such medicinally important plant is *Withania somnifera* (Ashwagandha), also known as “Indian Ginseng” or “Indian Winter cherry”, which is being used since ancient civilization as Ayurvedic rasayana [9]. It exhibits pleotropic effects in biological systems including neuroprotective, anti-inflammatory, immune-modulatory, and antitumor activity among others [10]^.^ Different parts of this plant have been used in many Ayurvedic formulations, most common being the root of Ashwagandha which contains many biologically active components such as alkaloids (isopelletierine, anahygrine etc.), saponins and steroidal lactones (withanolides and withaferins) [11].

**Fig. 1:**
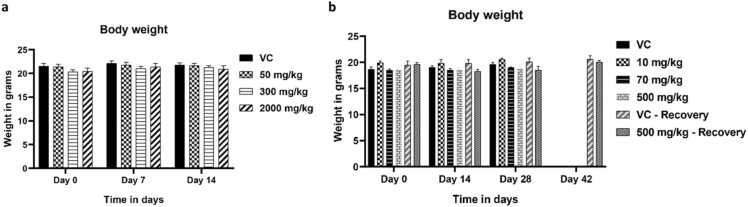
**: a.** Body weight of mice in acute toxicity. **b.** Body weight of mice in repeat dose toxicity. All values are represented as mean ± SEM.

Withaferin-A (WA), the principle active element of *Withania somnifera,* was first isolated from the leaves by Lavie and Yarden [12]. Other members of Solanaceae family also serve as source of WA [13]. WA has been shown to exhibit diverse therapeutic benefits against cancer, diabetes, psychological stress, oxidative stress, and immuno-modulation [3], [14]. It is also used as aromatic and flavouring agent [15]. These beneficial activities of WA is greatly attributed by its double bond and epoxide ring (structure of WA is given in supplementary file 1-Fig. 1) [16]. A recent study from our laboratory demonstrated that *ex-vivo* treatment of donor cells with WA could ameliorate the onset of Graft versus Host Disease (GvHD) in mouse model of allogeneic bone marrow transplantation by modulating Akt-mTOR signaling pathway [17]. Straughn et al., reported the efficacy of WA against SARS-CoV-2 infection, and suggested that WA could also be used for cancer patient with SARS-CoV-2 infection owing to its anticancer as well as anti-viral properties [18]. The anti-cancer activity of WA and its molecular mechanism have been extensively evaluated in several preclinical studies demonstrating that WA binds directly with various intracellular signaling molecules such as NFkB, Par-4 and STAT3 therefore it regulates proliferation, migration, invasion and metastasis [3], [19]. WA treatment increases the expression of autophagy markers and initiates autophagy induced cell death in in-vitro (MDA-MB- 231 and MCF-7 cell lines) and in tumor xenograft model of breast cancer [20]. Currently, in the era of cancer immunotherapy, adverse events such as cytokine release syndrome as a result of over activation of CD8^+^ T cells could be dose limiting. Gambhir et al., demonstrated that WA reduced the secretion of Th1 and Th2 cytokines and inhibited the mitogen-induced T-cell and B-cell proliferation. This immunosuppressive effect of WA was mediated through its binding with cysteine-62 residue of p50 and subsequent inactivation of the NF-κB pathway in T lymphocytes [21]. Thus WA could be a potential adjunct to cancer immunotherapy. In addition, WA has also exhibited protection against inflammatory conditions of liver, kidney and skin [22], [23], [24].

Despite its pharmacological actions, the translation of WA to the bedside has not been accomplished. Establishing the non-clinical toxicity and pharmacokinetics (PK) of a molecule is an important aspect of drug development, and provides vital insights about its safety and posology in humans. Several groups have worked extensively on understanding the toxicity of *Withania somnifera* extract [25], [26]. However, there is no data available on oral safety and toxicity of WA, which is the most biologically active component of *Withania somnifera*. With an aim to bring WA a step closer to the clinics, the current study evaluated the oral safety, toxicity and pharmacokinetics of WA in mice.

## 2 Material and methods

### 2.1 Chemicals and reagent

Withaferin-A and fluoximesterone was generous gift from Pharmanza herbal Pvt. Ltd, Gujrat, India. LC-MS grade ethyl acetate, ammonium acetate and acetonitrile, sulfosalicylic acid were used in the study. Milli-Q water was filtered additionally with a 0.22 µ filter paper using filter assembly.

### 2.2 Experimental animals

The study was initiated after approval from the Institutional Animal Ethics Committee of Advanced Centre for Treatment, Research and Education in Cancer (ACTREC), Tata Memorial centre (TMC), Mumbai, India (project no. 17/2020). Female BALB/c mice of 8–10 weeks old, weighing 20 ± 2 g were used for the study. Mice were housed in the small animal facility of ACTREC. Standard chow and water was given ad libitum*.* A 12 h light/dark cycle was maintained at 22–25° C temperature and humidity of approximately 50%. Mice were acclimatized for one week prior to experimentation. At the end of experiments all animals were humanely euthanized. All procedures were carried out as per the committee for the purpose of control and supervision of experiments on animals (CPCSEA) guidelines. The study was performed in accordance with the ARRIVE guidelines.

### 2.3 *In silico* absorption, distribution, metabolism, excretion and toxicity (ADMET) analysis

ADMET properties, as well as some physicochemical properties and medicinal chemistry friendliness of WA can be predicted by ADMETlab2.0 online tool (https://admetmesh.scbdd.com/). Briefly, the 2D structure of WA was retrieved from PubChem database and the SMILES format was generated by submitting the structure of WA to SwissADME tool (http://www.swissadme.ch/index.php). This SMILES was then acquiesced to ADMETlab2.0 for ADMET analysis.

### 2.4 Prediction of toxic hazards

Toxtree (v3.1.0) was used to assess the toxic hazards of WA by applying decision tree approach. Toxtree decision tree was employed for pertaining the Verhaar scheme, skin irritation and corrosion prediction, eye irritation and corrosion prediction, Benigni/Bossa rule base (for mutagenicity and carcinogenicity), skin sensitization alerts, START biodegradability, cytochrome P450-mediated drug metabolism, DNA binding alerts, protein binding alerts, etc. It can estimate the threshold of toxicological concern (TTC) of the compound or their possible toxicity. The molecule site(s) labile to metabolism by cytochrome P450 isoform 3A4 was predicted by SMARTCyp reactivity model.

### 2.5 Acute toxicity

The acute oral toxicity study was conducted as per the Organisation for Economic Cooperation and Development (OECD) test guidelines 423 adopted on 17th December 2001. Animals were kept on 4 h of fasting before and after administering the dose. A single oral dose of 50 mg/kg, 300 mg/kg and 2000 mg/kg of WA suspended in 100 µl of 0.5% carboxymethylcellulose (CMC) in 1X phosphate buffer saline was administered to mice using oral gavage. Vehicle control group was administered only 100 µl of 0.5% CMC in 1X phosphate buffer saline. Animals were monitored individually in the initial 4 h at 30 min interval post dosing, intermittently during the first 24 h, and once a day thereafter up to 14 days for any sign of clinical toxicity. Mice weight were recorded on day 0, 7 and 14. Mice were sacrificed after 14 days and blood samples was collected for clinical biochemistry and hematological investigation. Vital organs were harvested for histopathology examination.

### 2.6 Sub-acute toxicity

Sub-acute toxicity or 28 days repeat dose toxicity study were performed in accordance with OECD guideline 407 adopted on 3 October 2008. Thirty female mice were divided into six groups (five mice per group); 1. Vehicle control (CMC), 2. 10 mg/kg treatment (low dose), 3. 70 mg/kg treatment (medium dose) 4. 500 mg/kg treatment (high dose), 5. Vehicle control (satellite/recovery groups) and 6. 500 mg/kg treatment (satellite/recovery groups). WA was administered in 100 µl of 0.5% carboxymethylcellulose (CMC) in 1X phosphate buffer saline daily for 28 days through oral gavage. Two satellite groups, after 28 days of dosing, were kept under observation for 14 more days to check the reversal or delayed onset of toxicity. All mice were observed every day for any clinical sign of toxicity. Mice weight were recorded on day 0,7,14 and 28. For the satellite groups weight were recorded again on day 42. At the end of the experiment, mice were sacrificed, blood samples were collected for biochemical, hematological investigation and vital organs were harvested for histopathology.

### 2.7 Biochemical evaluation

Following 400–50 μl of blood collection in clot activator tube, serum was harvested by centrifugation at 3000 rpm for 10 min. Liver function test (LFT) and renal function test (RFT) were done using Dimension EXL 200-Siemens (Germany) autoanalyzer.

### 2.8 Hematological analysis

Blood samples (100 μl) collected in EDTA tubes were subjected to complete blood count (CBC) analysis in ADVIA 2120i (USA) autoanalyser. Hemoglobin, RBC, total count, platelets and other blood parameters were analysed.

### 2.9 Percentage lymphocytes and neutrophil count

Manually, slides were prepared for counting the lymphocytes and neutrophils as per the protocol described by Hoppe et al., [27]. Briefly, blood smear was made on a glass slide using 10 µl of blood. Further, using the Wright stain, slides were stained and kept for air drying before counting. The cells were counted under microscope by two independent experts from the composite laboratory of ACTREC.

### 2.10 Histopathology

Immediately after sacrificing the mice, tissues for histopathology were collected in 10% formalin and sent to histopathology facility of ACTREC for the preparation and staining of sides. Further the slides were evaluated by two independent pathologist from our centre to investigate any possible signs of tissue toxicity.

## 3 Pharmacokinetics

### 3.1 Animals for PK study

Female BALB/c mice were used for the PK study. Mice were given single oral dose of 70 mg/kg of WA and their blood samples was collected in EDTA tubes at 0.083, 0.25, 0.5, 1, 2, 4, 6, 8, 12, 24 h (5 mice per time points). To another set of female BALB/c mice, intravenous injection (IV) of WA was given at dose of 10 mg/kg, blood samples were collected in EDTA tubes at 0.03, 0.17, 0.5, 1, 2, 4, 6, 8, 12, 24 h (5 mice per time points). Blood from both the groups (oral & IV) were centrifuged at 3000 rpm for 10 min at 4 °C. Plasma samples were collected and kept in − 80 °C until further analysis. The level of plasma WA were quantified by using LC-MS/MS as per the protocol mentioned in Supplementary file 1. The PK parameters were evaluated by non-compartmental analysis using Phoenix WinNonlin (CERTARA) software (version 8.3., NJ, USA). The absolute oral bioavailability (F) of WA was calculated using the equation….

F (%) = AUC_0-inf_(Oral)*Dose IV / AUC_0-inf_(IV)*Dose Oral *100

### 3.2 Mass spectrometry and chromatographic conditions

LC-MS/MS method was performed using the AB SCIEX QTRAP-4500 LC-MS/MS instrument, LC SHIMADZU Nexera X2 Micro LC. A Kinetex® 1.7 µm C18 100 Å (100 *3 mm, S/No. H20–111310, Batch No. XD-4475-YO) column was used for chromatographic separation. The mobile phase consisted of acetonitrile and 10 mM ammonium acetate in milli-Q water (60:40 v/v). Mass spectrometer was operated in the positive ion mode. The aqueous phase was eluted at a flow rate of 0.2 mL/min and the analytes were quantified in MRM mode using the following mass transitions i.e., WA - Q1/Q3 471.400/ 281.200 and fluoxymesterone, the internal standard (IS), -Q1/Q3 337.200/91.100. Quantitation was achieved by measurement of the peak area ratios of the drug to the internal standard. Data acquisition was performed with Analyst version 1.6.1 software.

### 3.3 PK parameters and statistical analysis

All biochemistry and haematological parameters were analysed using GraphPad Prism 8.0 software and expressed as mean ± SEM. One-way ANOVA followed by Tukey’s multiple comparison tests was done. *p* value < 0.05 was considered as statistically significant. All PK parameters were expressed as Mean ± SEM except T_max_ which is presented as median (range).

## 4 Results

### 4.1 *In silico* ADMET analysis

ADMET properties describes the therapeutic action of a molecule that depends on it reaching the target site in the body at an adequate amount. ADMETlab2.0 is a freely accessible that provides fast yet robust predictive data about the physicochemical properties, pharmacokinetics and drug-likeness of a compound. We utilized it to compute the physicochemical properties and bioavailability of WA. Detailed physicochemical and ADMET parameters are tabulated in Supplementary file 2. Concisely, topological polar surface area (TPSA) of WA falls in the acceptable zone of 0–40 whereas logS (aqueous solubility) was found to be slightly lower and logP (partition coefficient) was moderately higher than the optimum range. WA obeys Lipinski, Pfizer and Golden triangle rule that suggests the drug-likeness of a molecule. Absorption profile showed that WA is highly gastro-intestinal (Caco2 and MDCK) permeable and also acts as a strong P glycoprotein -inhibitor with a probability of 0.994. Distribution data outlined the plasma protein binding (PPB) and fraction unbound (Fu) of WA were 84.60% and 4.713% respectively. However, the probability of blood brain barrier (BBB) permeability is 0.725. Metabolism profile explained WA to be a CYP3A4 substrate with a probability of 0.895. WA showed high clearance of 16.18 mL/min/kg that was computed from the excretion profile of the ADMET analysis. The ADMET prediction suggest very low probability of being hERG (human Ether-a-go-go-Related Gene) blocker. The human hepatotoxicity of WA were estimated to be very low, similarly oral toxicity in rat were also predicted to be scarce. WA also determined to be noncorrosive to the eye.

### 4.2 Computation of toxic hazards

Cytotoxicity by CYP450-mediated drug metabolism for WA predicted 4 sites of metabolism viz. epoxidation and aliphatic hydroxylation. START biodegradability and Verhaar scheme explained WA to be a persistent chemical and possess unspecific reactivity based on its structural annotation. The presence of α,β-unsaturated carbonyls in WA explained the structural alerts for genotoxic carcinogenicity and S. typhimurium mutagenicity computed by Benigni/Bossa rule base (for mutagenicity and carcinogenicity). The protein binding and DNA binding alerts for Michael acceptor and SN2-nucleoplilic aliphatic substitution were also identified.

### 4.3 Acute toxicity

WA treated group did not show any clinical symptoms of toxicity compared to the vehicle control group. There was no visible sign of toxicity in any animal in the WA group, and none of the animals died. Based on these findings, WA may be classified as GHS (Globally Harmonized System) category 5 (LD_50_ >2000 mg/kg body weight) compound as per OECD Guideline No. 423, December 2001.

### 4.4 Sub-acute toxicity (28 days repeat dose toxicity)

Sub-acute toxicity were performed at dose of 10, 70and 500 mg/kg/day. Mice were dosed daily for 28 days. All animals were alive until necropsy in both the test group and the recovery (satellite) group. None of the animals in the test or recovery groups showed any visible signs of toxicity. These results suggest that oral administration of WA to mice every day for 28 days is safe and well tolerated. Therefore, 500 mg/kg oral WA shall be considered as No-Observed Adverse Effect Level (NOAEL) dose as per OECD Guideline No. 407, October 2008.

### 4.5 Body weight

Upon oral WA administration, mice in both acute toxicity as well as in sub-acute toxicity studies did not show any changes in body weight compared to their respective controls (Fig. 1a-b).

### 4.6 Serum biochemistry

Serum RFT and LFT parameters were investigated. RFT parameters included blood urea nitrogen (BUN), uric acid (URCA) and creatinine (CREA). LFT parameters included total protein (TP), albumin (ALB), alkaline phosphatase (ALP), total bilirubin (TB), aspartate aminotransferase (AST) and alanine aminotransferase (ALT). In acute and sub-acute toxicity, no changes were observed in LFT and RFT parameters in WA treated groups compared to their respective control groups (Table 1a, Table 1b).

**Table 1a:** Effect of orally administered Withaferin-A on RFT and LFT parameters in acute toxicity.

Test	Unit	VC	50 mg/kg	300 mg/kg	2000 mg/kg
**TP**	g/dl	5.9 ± 0.5	4.9 ± 0.4	5.4 ± 0.1	5.4 ± 0.1
**ALB**	g/dl	1.0 ± 0	1.1 ± 0	1.1 ± 0	1.1 ± 0
**ALP**	U/L	105.0 ± 8.5	103.3 ± 6.3	115.6 ± 6.3	130.6 ± 9.1
**TB**	mg/dl	0.1 ± 0	0.1 ± 0	0.1 ± 0	0.1 ± 0
**AST**	U/L	118.0 ± 18.2	107.3 ± 17.8	134.3 ± 22.8	121.3 ± 38.4
**ALT**	U/L	40.3 ± 2.7	47.7 ± 7.8	49.7 ± 4.4	45.7 ± 4.9
**BUN**	mg/dl	46.0 ± 1.5	45.0 ± 3.6	51.0 ± 2.1	56.3 ± 5.5
**URCA**	mg/dl	2.8 ± 0.6	2.8 ± 0.4	2.6 ± 0.1	2.8 ± 0.6
**CREA**	mg/dl	0.3 ± 0	0.2 ± 0.1	0.3 ± 0.1	0.3 ± 0.04

All values are represented as Mean ± SEM. TP: total protein, ALB: albumin, ALP: alkaline phosphatase, TB: total bilirubin, AST: aspartate aminotransferase, ALT: alanine aminotransferase, BUN: blood urea nitrogen, URCA: uric acid, CREA: creatinine

**Table 1b:** Effect of orally administered Withaferin-A on RFT and LFT parameters in sub-acute toxicity.

Test	Unit	VC	10 mg/kg	70 mg/kg	500 mg/kg	VC Recovery	500 mg/kg Recovery
**TP**	g/dl	4.9 ± 0.3	5.1 ± 0.0	4.9 ± 0.5	4.8 ± 0.4	5.3 ± 0.1	5.5 ± 0.1
**ALB**	g/dl	1.6 ± 0.1	1.4 ± 0.0	1.5 ± 0.4	1.7 ± 0.1	1.2 ± 0.2	1.3 ± 0.3
**ALP**	U/L	145.3 ± 8.0	123.8 ± 3.7	138.2 ± 6.2	138.6 ± 11.5	121.6 ± 3.9	133.4 ± 4.5
**TB**	mg/dl	0.1 ± 0.0	0.2 ± 0.0	0.3 ± 0.0	0.1 ± 0.0	0.2 ± 0.0	0.2 ± 0.1
**AST**	U/L	185.5 ± 13.8	181.4 ± 5.2	169.8 ± 31.9	162.4 ± 19.0	158.0 ± 9.4	173.4 ± 5.1
**ALT**	U/L	48.8 ± 5.9	42.4 ± 2.0	43.8 ± 4.7	46.4 ± 1.4	41.0 ± 1.7	51.0 ± 6.6
**BUN**	mg/dl	46.5 ± 5.9	50.4 ± 2	55.2 ± 2.3	50.6 ± 5.7	49 ± 2.0	45.6 ± 5.2
**URCA**	mg/dl	2.0 ± 0.3	2.4 ± 0.2	1.8 ± 0.1	1.2 ± 0.2	1.9 ± 0.3	2.1 ± 0.4
**CREA**	mg/dl	0.4 ± 0.0	0.3 ± 0.0	0.4 ± 0.0	0.5 ± 0.0	0.3 ± 0.1	0.4 ± 0.1

All values are represented as Mean ± SEM. TP: total protein, ALB: albumin, ALP: alkaline phosphatase, TB: total bilirubin, AST: aspartate aminotransferase, ALT: alanine aminotransferase, BUN: blood urea nitrogen, URCA: uric acid, CREA: creatinine

### 4.7 Hematological parameters

We analyzed the CBC and differential counts in acute and sub-acute toxicity animals following oral administration of WA. At any given acute toxicity dose none of the CBC parameters were altered, barring a non-significant dose dependent decrease in total WBC count (Table 2a). Hematological parameters in sub-acute toxicity also did not show any changes at any dose level tested compared to control groups (Table 2b).

**Table 2a:** Effect of orally administered Withaferin-A on hematological parameters in acute toxicity.

Test	Unit	VC	50 mg/kg	300 mg/kg	2000 mg/kg
**WBC**	10^3^/µl	5.1 ± 0.9	4.8 ± 0.5	3.5 ± 1.2	2.3 ± 0.5
**RBC**	10^6^/µl	9.9 ± 0.1	9.3 ± 0.1	9.22 ± 0	9.3 ± 0.2
**Hb**	g/dl	14.6 ± 0.1	13.7 ± 0.3	13.6 ± 0.1	13.6 ± 0.2
**HCT**	%	49.2 ± 0.3	46.1 ± 0.2	47.5 ± 0.3	46.3 ± 1.1
**MCV**	fl	49.4 ± 0.4	49.5 ± 0.7	50.9 ± 0.4	50.0 ± 0.4
**MCH**	pg	14.6 ± 0.1	14.7 ± 0.3	14.8 ± 0.1	14.7 ± 0.1
**MCHC**	g/dl	29.6 ± 0.1	29.9 ± 0.4	29.0 ± 0.1	29.4 ± 0.2
**PLT**	10^3^/µl	839.3 ± 28.2	759.3 ± 35.4	782.0 ± 49.1	710.3 ± 51.5
**NEUT**	%	14 ± 2	15 ± 5	12 ± 2	18 ± 7
**LYMPH**	%	81 ± 2	81 ± 5	83 ± 2	77 ± 9

All values are represented as Mean ± SEM. WBC: white blood cell count, RBC: red blood cell count, Hb: hemoglobin, HCT: hematocrit, MCV: mean corpuscular volume, MCH: mean corpuscular hemoglobin, MCHC: mean corpuscular hemoglobin concentration, PLT: platelet, NEUT: neutrophils, LYMPH: lymphocytes.

**Table 2b:** Effect of orally administered Withaferin-A on hematological parameters in sub-acute toxicity.

Test	Unit	VC	10 mg/kg	70 mg/kg	500 mg/kg		VC Recovery	500 mg/kg Recovery
**WBC**	10^3^/µl	3.7 ± 0.3	4.1 ± 0.4	3.4 ± 0.5	3.9 ± 0.4		3.8 ± 1.0	3.9 ± 0.4
**RBC**	10^6^/µl	8.5 ± 0.2	8.1 ± 0.2	8.9 ± 0.2	9.2 ± 0.3		8.2 ± 0.2	7.5 ± 0.2
**HGB**	g/dl	13.4 ± 0.3	11.9 ± 0.1	13.3 ± 0.3	13.7 ± 0.5		12.4 ± 0.2	11.2 ± 0.2
**HCT**	%	44 ± 0.5	40 ± 0.7	44.6 ± 1	45 ± 1		42 ± 0.8	39 ± 0.7
**MCV**	fl	50.8 ± 0.4	49.7 ± 0.4	50.1 ± 0.4	49.3 ± 0.5	51 ± 0.4		50.7 ± 0.7
**MCH**	pg	13.6 ± 0.8	11.8 ± 2.7	15 ± 0.2	14.9 ± 0.1		15.1 ± 0.2	14.9 ± 0.2
**MCHC**	g/dl	30.1 ± 0.1	29.3 ± 0.2	29.9 ± 0.3	30.3 ± 0.5		29.7 ± 0.3	29.3 ± 0.1
**RDW**	%	11.7 ± 0.4	11.7 ± 0.3	11.6 ± 0.2	11.6 ± 0.3		12.2 ± 0.1	12.4 ± 0.3
**PLT**	10^3^/µl	649.0 ± 17.2	639.6 ± 33.4	666.5 ± 18.6	684.7 ± 52.9		678.8 ± 22.2	652.0 ± 23.3
**NEUT**	%	13 ± 3	16 ± 5	14 ± 1	16 ± 3		15 ± 4	14 ± 2
**LYMPH**	%	82 ± 2	79 ± 5	81 ± 2	80 ± 4		80 ± 3	82 ± 3

All values are represented as Mean ± SEM. WBC: white blood cell count, RBC: red blood cell count, Hb: hemoglobin, HCT: hematocrit, MCV: mean corpuscular volume, MCH: mean corpuscular hemoglobin, MCHC: mean corpuscular hemoglobin concentration, LT: platelet, NEUT: neutrophils, LYMPH: lymphocytes

### 4.8 Histopathology

**Fig. 2:**
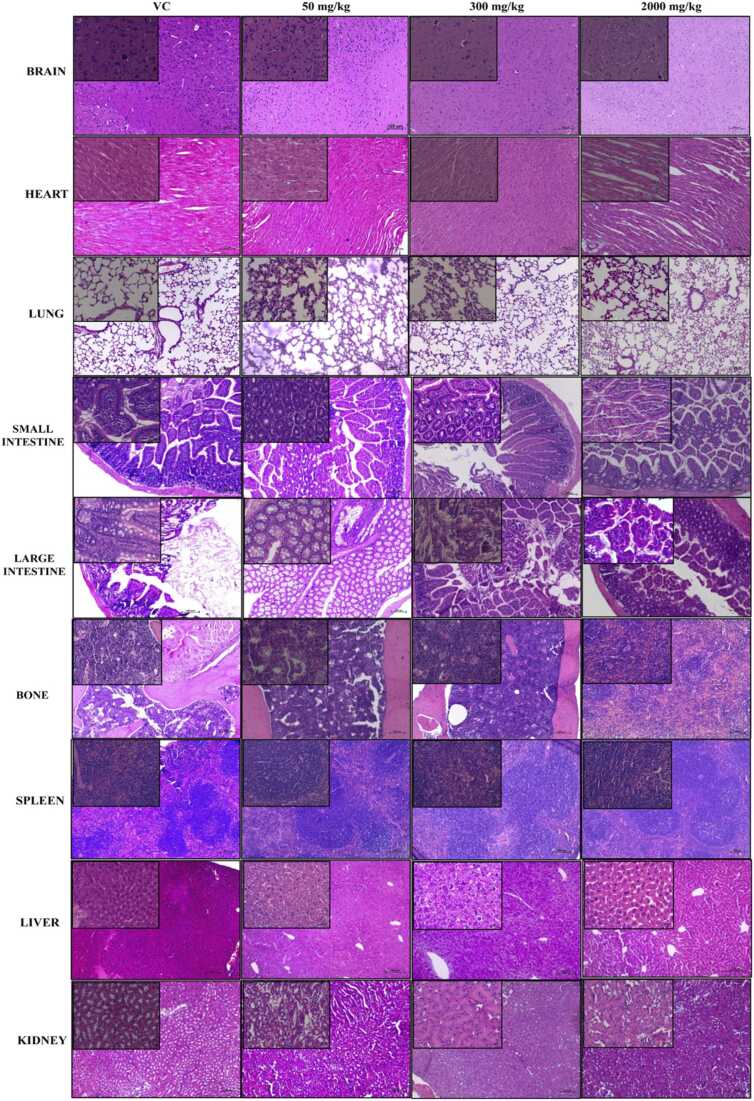

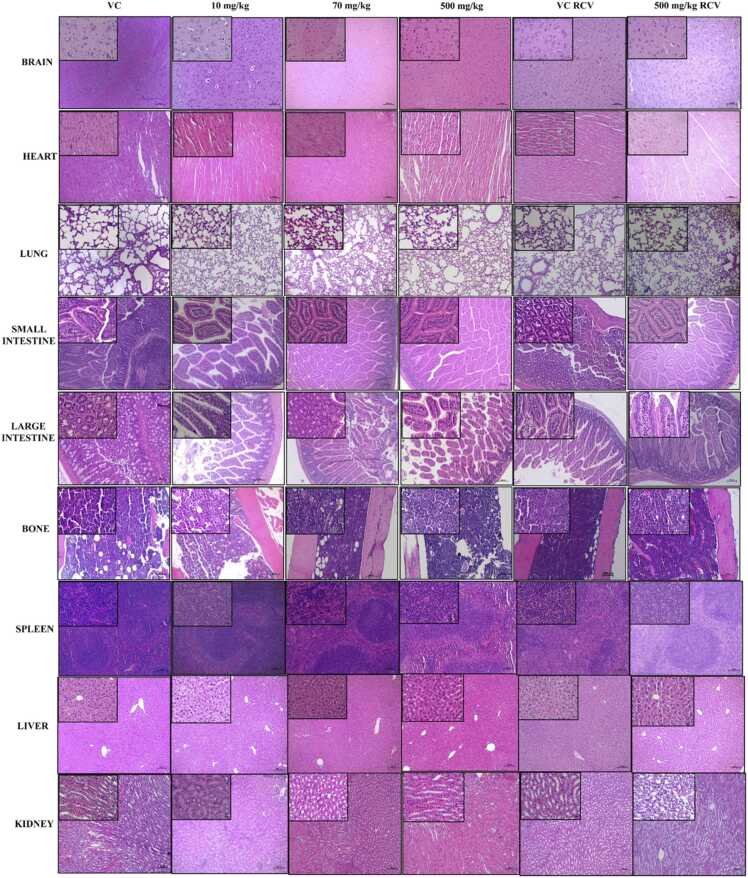
**: a.** Histopathology of different organs of control and WA administered mice in acute toxicity. **b.** Histopathology of different organs of control and WA administered mice in sub-acute toxicity. H and E magnification × 100 and × 400 (Inset). VC: vehicle control, RCV: recovery.

Upon histopathological observation of vital organs such as brain, heart, lungs, liver, kidney, spleen, bone, small and large intestine, none of them showed any sign of drug induced toxicity in acute as well as in sub-acute toxicity studies (Fig. 2a and b).

### 4.9 Pharmacokinetics of WA

**Fig. 3:**
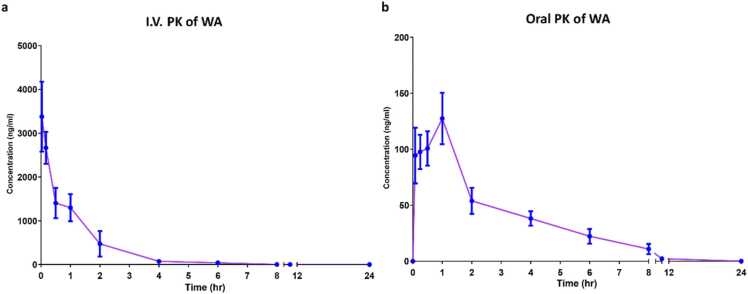
**: a.** Mean plasma concentration versus time curve for WA after IV administration. **b.** Mean plasma concentration versus time curve for WA after oral administration.Values are expressed as means ± SEM (n = 5 mice/time points).

PK samples were analyzed for WA levels using a validated LC-MS/MS technique. The validation parameters of the method are shown in supplementary file 1. Maximum plasma concentration (C_max_) were found to be 3996.9 ± 557.6 ng/mL and 141.7 ± 16.8 ng/mL for IV and oral doses respectively. The median T_max_ following oral administration was 0.5 h. The mean plasma concentration-time curves of WA are illustrated in Fig. 3(a-b) and the PK parameters are shown in Supplementary file 1-table 8. Further, the percentage bioavailability of WA was found to be 1.8%.

### 4.10 Discussion

Phytochemicals have shown benefit in prevention of diseases and their progression [28], although, the application of most phytochemicals is limited by poor bioavailability and sometimes toxicity [29]. WA, a steroidal lactone obtained from Ashwagandha, is known for its therapeutic effects in traditional Indian medicine against cancer, hepatitis, Alzheimer’s disease, among others [30], [31].

In the current study, for the first time we carried out a comprehensive toxicity evaluation of pure WA. Earlier, toxicity studies have been carried out on *Withania somnifera extract* having a mixture of withanosides and withanolides, which may not truly reflect the toxicity of its individual components. Therefore profiling the toxicity and pharmacokinetics of WA, one of the most promising phytomedicine, is desirable.

Initially, we carried out *in silico* studies to predict the ADMET properties of WA. The parameters obtained from the ADMET analysis based on the structural chemistry of the drug predicted good safety and tolerability profile in mice which were corroborated in the in vivo toxicity studies. The current study was carried out using female mice, because females are generally more sensitive to drug related toxicity [32]. Based on the results of acute toxicity study, WA can be classified as a Category 5 compound, implying ‘relatively safe’, as per the globally harmonized classification System (GHS) of drugs (OECD guideline 423). The observed NOAEL dose through repeat dose toxicity experiment was demonstrated to be 500 mg/kg. Considering the favourable toxicity profile, the current study will aid future development of pure WA for clinical use, highlighting the significance of the study.

While to the best of our knowledge this is the first study to conclusively demonstrate the toxicity profile of WA in mice, Prabhu et al., reported the safety of a hydro alcoholic extract of Withania somnifera root (WSR) up to 2000 mg/kg in acute and sub-acute toxicity studies in rats. No significant hematological, biochemical and histological changes were observed in Wister rats at these doses [25]. Similar results were reported by Shruti et al., using methanolic extract of WSR containing 4.5% of WA [26]. Despite the high doses of WSR used in these studies, the absolute dose of WA present in these extracts was significantly less than that used in our study, although WSR will have other withanoloids whose toxicity profile vis-à-vis WA is yet to be studies. Nevertheless, all these studies taken together clearly demonstrate the safety of WA in rodents at high doses. Of course, oral administration may not be a true reflection of a drug’s safety, particularly if it has low oral bioavailability. Therefore, the reports from Sharada et. al., and Shohat et. al., assumes significance in this context as both groups reported LD_50_ of WA at sub-100 mg/kg dose in acute toxicity studies following intraperitoneal (IP) administration (80 mg/kg and 54 mg/kg respectively) [14], [33]. The difference in LD_50_ between the two studies is perhaps attributable to differences in environment, method of isolation and species variation between herbs grown in India and Israel [33].

Pharmacokinetic study showed low oral bioavailability of WA which also corroborated the outcome of *in silico* analysis. *In silico* analysis predicted a high first-pass effect due to extensive metabolism by CYP3A4 in the liver, while absorption across the GI tract itself was not expected to be low given a high Caco-2 and MDCK-permeability. Dai et al., demonstrated rapid metabolism of WA in liver microsomes which explains the first-pass effect [34]. True to this phenomena, in our study the peak concentration following oral administration was achieved rapidly but was less than 1% of the maximum concentration achieved through intravenous route (normalized for dose). Similar findings were also reported by Patil et al., who observed a rapid (T_max_=20 min) but low C_max_ of 16.69 ng/mL following oral administration of 1000 mg/kg of an aqueous root extract of *Withania somnifera* [35]*.* Surprisingly, Dai et al., reported an exceptionally high average C_max_ of 619 ng/mL with a 10 mg/kg oral dose of WA. Further, they reported oral bioavailability of approximately 32%. The difference in PK parameters could be possibly explained by the vehicles used in the two studies. We administered oral WA in CMC which forms drug suspension while Dai et al. formulated WA in ethanol–solutol HS 15–distilled water (10:5:85, v-v:v). They observed an extremely rapid oral absorption (T_max_= 6 min) which could have possibly saturated the CYP enzymes, leading to high C_max_, and consequently a higher AUC. Recently, Modi et. al., carried out the PK study of withanosides (withanoside IV, withanoside V) and withanolides (WA, withanolide A, 12-Deoxy-withastramonolide) by administering 500 mg/kg of a *Withania somnifera extract* in rats. They reported the T_max_ of 13, 124, 157 and 7 ng/mL for withanoside IV, WA, 12-deoxy-withastramonolide and withanolide A respectively. Other constituents such as withanone and withanolide B were reported below lower limit of quantification. The PK pattern of WA in the extract reported in this study corroborates our finding [36].

With the increasing use of herbal medicine in modern era and considering their poor bioavailability, there is surge in research on bioenhancers. Bioenhancers have the potential to increase the bioavailability of drugs by modulating their first pass metabolism or membrane permeation [37]. Results of our in-silico study and earlier report of Dai and colleagues suggest that WA may undergo strong first pass effect as it is a substrate of P-glycoprotein and microsomal cytochromes P450 3A4 (CYP3A4) [34]. Piperine being a natural bioenhancer, is known to inhibit P-glycoprotein and CYP3A4 and prevent the first pass metabolism of drugs, thereby increasing their bioavailability [38]. Thus, combining WA with piperine may be desirable when higher concentration of WA is required for its in-vivo biological activity.

Earlier in a phase I clinical trial using standardised *Withania somnifera extract*, our group demonstrated the safety of WA up to 216 mg/day in patients with osteosarcoma [10]. However, the PK of WA could not be studied owing to low sensitivity of the HPLC method employed for bioanalysis. None of the pharmacokinetic samples collected in this study had WA levels above the lower limit of quantitation of 50 ng/mL. Thus, WA seems to have uniformly low bioavailability across species. Thus, co-administration with a bioenhancer should be explored in future pharmacokinetic studies of WA.

### 4.11 Conclusion

Our results suggest that WA is safe at doses of 2000 mg/kg and 500 mg/kg in acute and repeat dose toxicity respectively, albeit with low oral bioavailability. In future, development of newer, better bioavailable formulations of WA, perhaps in combination with bioenhancers, should be considered to exploit its therapeutic potential.

## Funding

The study was funded through the Intramural Annual Scientific Funding of Tata Memorial Centre, India.

## CRediT authorship contribution statement

**Saurabh Kumar Gupta:** Writing – original draft, Conceptualization, Data curation, Formal analysis, Methodology, Investigation, Validation, Visualization. **Shraddha Jadhav:** Data curation, Formal analysis, Validation. **Dievya Gohil:** Data curation, Investigation. **Girish Ch. Panigrahi:** Data curation, Investigation**. Rajiv Kumar Kaushal:** Data curative, Investigation, Validation**. Khushboo Gandhi:** Validation, Visualization**. Anand Patil:** Formal analysis, Software. **Preeti Chavan:** Resources, Validation**. Vikram Gota:** Conceptualization, Methodology, Investigation, Formal analysis, Resources, Supervision, Project administration, Funding acquisition, Writing – review & editing.

## Declaration of Competing Interest

The authors declare that they have no known competing financial interests or personal relationships that could have appeared to influence the work reported in this paper.
