# Supplementary material for: Safety, toxicity and pharmacokinetic assessment of oral Withaferin-A in mice
Source: Toxicol Rep. 2022 May 18;9:1204–12. doi: 10.1016/j.toxrep.2022.05.012 (PMC9742883; doi:10.1016/j.toxrep.2022.05.012)
Supplement: Supplementary file 1 — Supplementary material [file mmc1.docx]

**Supplementary file 1:**

**Results:**

**Supplementary fig 1. Structure of Withaferin-A. (Adapted from pubchem)**

**
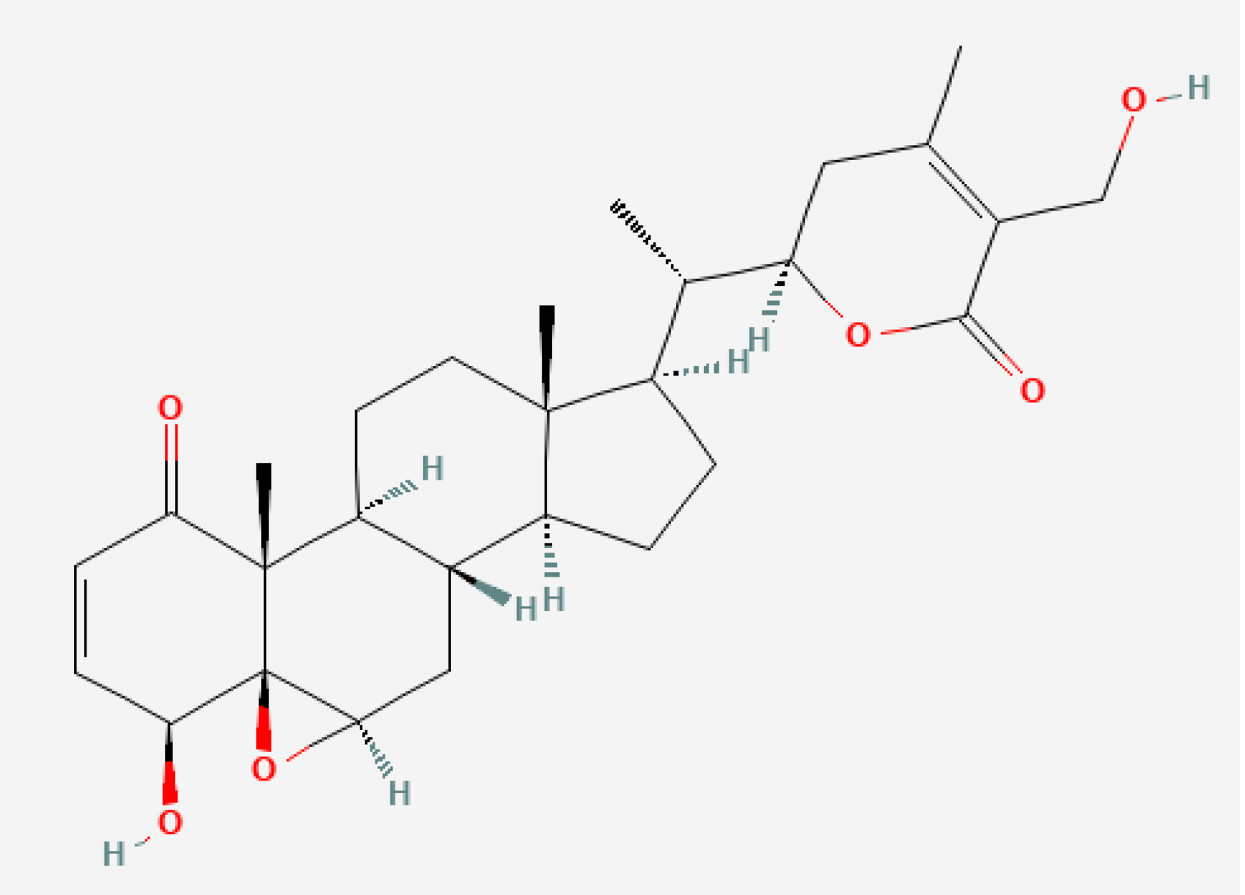
**

**Bioanalytical method development and validation**

**Standard solutions for Pharmacokinetics study:** Using separate weighing, Stock solution of Withaferin-A (WA) and fluoxymesterone were prepared for standard calibration curve and quality control (QC) samples. Dimethyl sulfoxide (DMSO) was used to prepare stock solution of WA and fluoxymesterone at concentration of 1 mg/mL, and stored at -20º C until further analysis.

**Sample preparation for Pharmacokinetics:** To 0.2 ml of plasma in a 1.5 ml eppendorf tube, 20µL of internal standard solution (20 µg/mL) was added, and the tubes were vortexed for 30 seconds. Followed by addition of 20µL of 4% sulfosalicylic acid, vortexed for 1min. For extraction, 1 ml of 100% ethyl acetate was added, samples were vortexed for 1 min and centrifuged at 13000 rpm for 15 minutes. The upper layer was transferred to a clean test tube and evaporated under nitrogen gas. The residue was reconstituted in 100µL of 60% acetonitrile in milli-Q water, vortexed and centrifuged at 13000 rpm for10 minutes and 5 µL of the final clear solution was injected into the LC-MS/MS system.

**Calibration curves and quantitation for Pharmacokinetics:** Calibration samples of Withaferin-A were prepared in blank plasma. A series of standard solutions at concentrations of 1, 5, 10, 25, 50, 100, 500, 1000, 2500 and 5000 ng/mL were prepared by dilution of the standard solution in blank plasma to obtain different working solutions and stored at -20°C. Preparation procedure described above was followed for each sample and 5 µL of the final solution was injected into the column. Plasma drug concentrations of WA was determined based on the ratio of peak area of WA (x) to the internal standard (y) using linear regression to calculate the unknown concentration levels from the calibration curve.

**Mass Spectrometry conditions:** Optimized MS parameters for the detection of analyte and IS are listed in Table 1.

**Table 1: The optimized MS conditions for Multiple Reaction Monitoring (MRM) mode.**

| **Ionization mode** | Positive ESI |
| --- | --- |
| **Ion source** | Turbo spray |
| **Curtain gas** | 40 |
| **Collision gas** | Medium |
| **Ion spray voltage** | 5500.00 |
| **Temperature (^0^C)** | 500.00 |
| **Ion source gas1 (GS1)** | 50 |
| **Ion source gas2 (GS2)** | 50 |

ESI; electrospray ionization.

**Pharmacokinetic method validation:** A 5-day validation analysis of WA was performed. The percent deviation (%DEV) and the relative standard deviation (%RSD) were calculated using Microsoft excel. The acceptance criteria for method validation were followed as per the bioanalytical method validation guidelines, May 2018.

**Selectivity:** The presence of possible disturbing endogenous peaks was examined on control human plasma samples obtained from the center’s blood bank. These samples were pretreated according to the sample preparation procedure without internal standard. Area of the interference peak should be ≤ 20% of peak area of the lower limit of quantification (LLOQ) for analyte and ≤ 5% for IS (table 2).

**Table 2: The average percentage Interference for analyte and IS**

| **Levels** | **Analyte area** | **IS area** | **% Interference for analyte** | **% Interference of IS** |
| --- | --- | --- | --- | --- |
| **Plasma blank** | 39 | 45 | 0.21 | 0.06 |
| **LLOQ** | 18732 | 78313 |  |  |

**Carryover:** Carry over in the blank sample following the high concentration standard should be ≤ 20% of the LLOQ for analyte and ≤ 5% for the internal standard.

**Linearity:** Calibration standards were used to construct calibration curves from which the concentrations of analytes in QC samples and in study samples were determined. The calibration model is accepted if percent accuracy was within ± 20% for limit of quantification (LOQ) and within ± 15% for all other standard concentrations (table 3).

**Table 3: The mean correlation coefficients of the linear regression analysis of calibration curve.**

| **Levels (ng/mL)** | **Mean** | **SD** | **%RSD (± 20%)** | **% Accuracy (85-115%)** |
| --- | --- | --- | --- | --- |
| **1** | 1.2 | 0.1 | 16.4 | 116.36 |
| **5** | 5.7 | 0.8 | 13.1 | 113.05 |
| **50** | 56.8 | 9.0 | 13.6 | 113.61 |
| **500** | 571.8 | 32.4 | 14.4 | 114.36 |
| **1000** | 1000.2 | 74.3 | 0.0 | 100.02 |
| **2500** | 2195.2 | 355.6 | -12.2 | 87.81 |
| **5000** | 5242.5 | 104.4 | 4.8 | 104.85 |

The calibration curves were constructed by plotting the ratio of WA peak areas to that of IS versus standard WA concentrations. The developed method demonstrated linearity in the concentration range of 1-5000 ng/mL in plasma. The regression equations for the calibration curve of WA in plasma was given by y=mx+c, where ‘y’ indicates the ratio of peak area of WA to IS and ‘x’ indicates WA concentration. Mean correlation coefficient (R^2^) of the calibration curve were 0.9970. **Acceptance criteria:** Non-zero calibrator should be within ± 15% RSD, except for LOQ for which it should be within ± 20% RSD. RSD: Relative standard deviation.

**Sensitivity (limit of quantitation):** Limit of detection (LOD) was determined using the signal-to-noise ratio by comparing the known concentrations of analyte to blank samples. Signal-to noise ratio of 3:1 produced by analyte concentration was accepted as the LOD. The LOQ is define as the lowest plasma concentration of the standard curve that could be quantified with acceptable accuracy, precision, and variability with acceptable accuracy and precision of **±** 20% (table 4).

**Table 4:** **The LOQ for Withaferin-A in extracted plasma**

| **Levels (ng/mL)** | **Average mean area** | **SD** | **%RSD (± 20%)** |
| --- | --- | --- | --- |
| 1 | 0.4129 | 0.004 | 0.97 |

The LOQ for WA in extracted plasma was 1 ng/mL with precision expressed as a %RSD of 0.97%. **Acceptance criteria:** The accuracy and precision should be within ± 20%. SD: Standard deviation, RSD: Relative standard deviation.

|  | Levels | Concentration (ng/mL) | Mean | SD | %RSD (± 15%) | % Accuracy (85-115%) |
| --- | --- | --- | --- | --- | --- | --- |
| **Intra-day** | LLOQQC | 1 | 1.1 | 0.11 | 13 | 113 |
|  | LQC | 5 | 5.6 | 0.60 | 11.1 | 111.1 |
|  | MQC | 500 | 497.7 | 132.07 | -0.457 | 99.543 |
|  | HQC | 2500 | 2640.6 | 113.18 | 5.622 | 105.622 |
| **Inter-day** | LLOQQC | 1 | 1.104 | 0.061 | 10.37 | 110.37 |
|  | LQC | 5 | 5.263 | 0.155 | 5.27 | 105.27 |
|  | MQC | 500 | 513.737 | 187.259 | 2.75 | 102.75 |
|  | HQC | 2500 | 2357.283 | 239.702 | -5.71 | 94.29 |

**Precision and Accuracy:** Using the same stock solutions and plasma batches, inter and intra-day precision and accuracy of analytical method were determined for WA (n=5 replicate for all quality control samples). The percent RSD of the assay were calculated. **Acceptance criteria**: Intra and inter-day precision & accuracy should be within ± 15% of nominal concentrations, except within ± 20% at LLOQ (table 5).

**Table 5: The intra and inter-day precision and accuracy for plasma samples.**

The intra and inter-day precision and accuracy for plasma samples are presented in Table 5. In plasma, quality control samples the mean precision were 5.36% and -3.64%, and accuracy were 105.36% and 96.36%. LLOQQC: Lower limit of quantification quality control, LQC: Lower quality control, MQC: Middle quality control, HQC: High quality control, SD: Standard deviation, RSD: Relative standard deviation.

**Recovery:** WA’s recovery was calculated by comparing the peak area obtained following extraction of known concentration of WA from plasma with that of the area obtained from the same concentration of WA in aqueous solution (table 6).

**Table 6: Recovery**

| Levels | Un-extracted area of analyte | Levels | Extracted area of analyte | % Recovery |
| --- | --- | --- | --- | --- |
| AQ- LLOQQC | 18411 | PLS- LLOQQC | 12050 | 65.45 |
| AQ-LQC | 65438 | PLS-LQC | 49210 | 75.20 |
| AQ-MQC | 301169 | PLS-MQC | 213488 | 70.89 |
| AQ-HQC | 1245622 | PLS-HQC | 777689 | 62.43 |

The overall mean recovery of Withaferin-A was 69.76 % after plasma extraction. AQ: Aqueous, PLS: Plasma, LLOQQC: Lower limit of quantification quality control, LQC: Lower quality control, MQC: Middle quality control, HQC: High quality control.

**Stability:** The stability of WA was assessed by analyzing samples under various conditions like auto-sampler, wet extract, dry extract, benchtop, freeze thaw, short term( 6hr) and long term (7^th^, 15^th^ day) stability and was checked the variation in the quality control samples respectively (table 7). **Acceptance criteria:** Percent RSD should be within ± 15% for quality control samples. RSD: Relative standard deviation.

**Table 7: stability**

| **Stability** | **Levels** | **Concentration (ng/mL)** | **Mean** | **%RSD (± 15%)** |
| --- | --- | --- | --- | --- |
| Auto-sampler | LQC | 5 | 4.50 | 0.25 |
|  | HQC | 2500 | 2450 | 0.51 |
| Wet extract | LQC | 5 | 4.95 | 1.05 |
|  | HQC | 2500 | 2438 | 1.25 |
| Dry extract | LQC | 5 | 5.05 | 1.05 |
|  | HQC | 2500 | 2499 | 0.85 |
| Benchtop | LQC | 5 | 4.95 | 1.24 |
|  | HQC | 2500 | 2581 | 0.85 |
| Freeze thaw | LQC | 5 | 5.05 | 1.25 |
|  | HQC | 2500 | 2492 | 2.5 |
| Short and long term | LQC | 5 | 5.12 | 2.51 |
|  | HQC | 2500 | 2483 | 2.51 |

**Fig 2: Chromatogram of WA and Fluoxymesterone.**

**
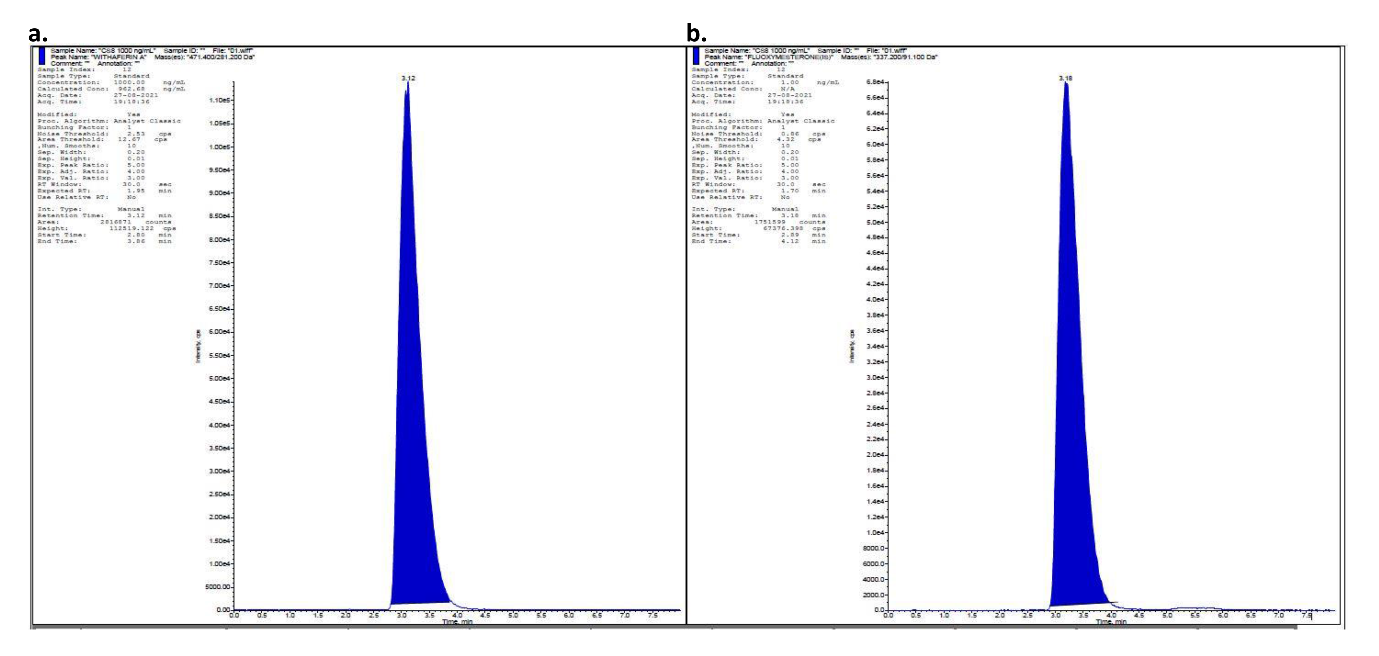
**

**Chromatograms of plasma spiked with WA and the IS Fluoxymesterone.** **a)** Withaferin-A and **b)** Fluoxymesterone eluted with high resolution within the run time of 8 minutes at 3.12 min and 3.18 min, respectively.

**Table 8.** **Pharmacokinetic parameters in mice after IV and oral administration of Withaferin-A.**

| **Parameters** | **I.V. WA (10 mg/kg)** | **Oral WA (70 mg/kg)** |
| --- | --- | --- |
| C_max_ (ng/mL) | 3996.9 ± 557.6 | 141.7 ± 16.8 |
| T_max_ (h) | - | 0.5 (0.25-1.0) |
| t_1/2_ (h) | 0.6 ± 0.4 | 2.7 ± 0.4 |
| AUC 0-∞ (ng/mL*h) | 3509.8 ± 302.4 | 436.1 ± 60.9 |
| CL(L/hr/kg) | 2.9 ± 0.2 | 171.1 ± 19.5 |
| V (L/kg) | 5.2 ± 1.2 | 656.6 ± 107.7 |
| % F | - | 1.8 |

All data presented in Mean ± SEM, T_max_ presented as median ± range. AUC: Area under the concentration–time curve; t_1/2_: half‐life; T_max_: time to peak concentration; CL: clearance; V: volume of distribution; C_max_: peak concentration; F: bioavailability.
